# *ChemSpaX*: exploration of chemical space by automated functionalization of molecular scaffold†

**DOI:** 10.1039/d1dd00017a

**Published:** 2022-01-06

**Authors:** Adarsh V. Kalikadien, Evgeny A. Pidko, Vivek Sinha

**Affiliations:** Inorganic Systems Engineering, Department of Chemical Engineering, Faculty of Applied Sciences, Delft University of Technology Van der Maasweg 9 2629 HZ Delft The Netherlands E.A.Pidko@tudelft.nl V.Sinha@tudelft.nl

## Abstract

Exploration of the local chemical space of molecular scaffolds by post-functionalization (PF) is a promising route to discover novel molecules with desired structure and function. PF with rationally chosen substituents based on known electronic and steric properties is a commonly used experimental and computational strategy in screening, design and optimization of catalytic scaffolds. Automated generation of reasonably accurate geometric representations of post-functionalized molecular scaffolds is highly desirable for data-driven applications. However, automated PF of transition metal (TM) complexes remains challenging. In this work a Python-based workflow, *ChemSpaX*, that is aimed at automating the PF of a given molecular scaffold with special emphasis on TM complexes, is introduced. In three representative applications of *ChemSpaX* by comparing with DFT and DFT-B calculations, we show that the generated structures have a reasonable quality for use in computational screening applications. Furthermore, we show that *ChemSpaX* generated geometries can be used in machine learning applications to accurately predict DFT computed HOMO–LUMO gaps for transition metal complexes. *ChemSpaX* is open-source and aims to bolster and democratize the efforts of the scientific community towards data-driven chemical discovery.

## Abstract 2

This work introduces *ChemSpaX*, an open-source Python-based tool for automated exploration of chemical space of molecular scaffolds with a special focus on transition-metal complexes.

## Graphical abstract

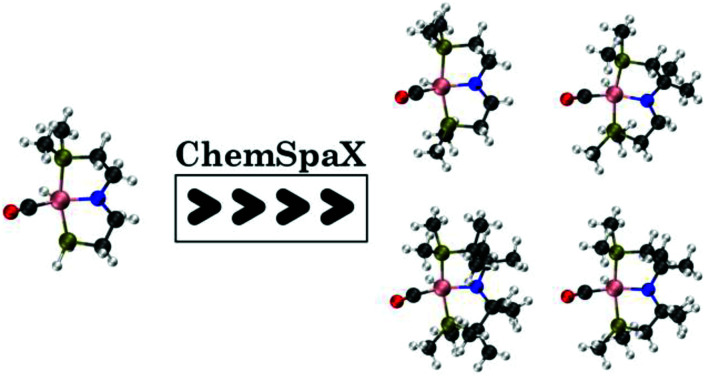

## 1 Introduction

Chemical research has been driven by the ability and the need to create molecular scaffolds with desired (bio)chemical functions. Experimental chemistry, largely guided by intuition, chemical knowledge, and serendipity has been reasonably successful in discovering functional molecular scaffolds which can be improved further. For example, reactive catalytic scaffolds are decorated with diverse functional groups *via* post-functionalization (PF) to explore their activity and stability, and devise possible strategies for improvement.^1–3^ Although *in vitro* functionalization can reveal chemical design principles that underlie high activity, selectivity and stability, it is time and resource intensive. Computational molecular design has emerged as particularly promising in this regard, thanks to recent advances in quantum chemical methods and high performance computing.^4–11^ 3D geometric information such as xyz coordinates and crystal structures of several catalytic scaffolds are known in the literature and databases. Using these known scaffolds, high-throughput computational methods can guide towards screening of highly effective PF strategies by systematically exploring geometries in the local chemical space of a given scaffold.^12^

The chemical space is vast and a global exploration is difficult.^13,14^ Therefore, machine learning (ML) and other cost-effective computational methods are attractive solutions to navigate the chemical space in the search of novel molecules and materials.^15^ A data-driven statistical approach, rooted in quantum and statistical mechanics (QM and SM) is needed to explore and understand the chemical space.^16^ Such an approach is strongly dependent on the availability of trustworthy structure property databases (SPDBs). For (small) organic molecules, reliable data sets such as the GDB datasets (11, 13 and 17) exist which are being used for diverse data-driven chemical discovery applications.^17–24^ In contrast to small organic molecules, development of data-driven approaches have proven more challenging for transition metal complexes (TMCs). TMCs are often used as *bio-inspired* homogeneous catalysts which account for over 15% of all industrial catalytic processes and enable key catalytic transformations such as for pharmaceuticals, fine chemicals, and energy applications.^25–32^ Recent studies have revealed the promise of data-driven quantum chemical methods to understand structure–function relations in TMCs.^33–38^ Availability of structure–property data from QM calculations and/or experiments is central to the success of data-driven chemical approaches. SPDBs of homogeneous catalysts are currently not available.^31^ SPDBs with a dense representation of the chemical space of catalytic scaffolds can help discover design principles leading to the development of sustainable catalytic systems for various applications.^31,39–44^ Representations of the molecular structure is of high importance in SPDBs. Several approaches have been developed in this regard recently. For organic molecules string based SMILES representation is a popular and effective approach to encode the molecular structure.^45,46^ Generally SMILES encoding does not work well for TMCs and generation of 3D geometries using SMILES is an active research area.^47,48^

Low dimensional encoding of molecular representations of TMCs, while certainly desirable for ML applications, co-exists with the need to know the 3D geometry due to high structural sensitivity in catalysis. Therefore, automated approaches to rapidly generate accurate 3D molecular representations are also needed. Some automated tools have recently been developed to generate molecular geometries of TMCs, such as MolSimplify, Aarontools, stk and Molassembler.^35,49–55^ Although they are all open-source and pythonic, each have their own advantages and disadvantages. In Aarontools, *substitute.py* offers a similar functionality to *ChemSpaX*. However, the added substituents are only optimized by minimizing the Lennard-Jones (LJ) energy. In *ChemSpaX* we use a force-field based approach, which is more extensive compared to minimizing the LJ energy. The stk package is topology based and each structure needs to be built from the ground up. To our best knowledge, the post-functionalization of an already existing scaffold has not been implemented. Additionally, in their *MetalComplex* topology graphs, stk currently only handles mono- and bidentate coordination geometries, which limits its general applicability. In Molsimplify, it should be possible to implement automated functionalization using the ‘*ligand decoration*’ or ‘*custom core*’ functionality when building a structure as presented in their tutorials.^56^ However, high-throughput functionalization of an already existing scaffold in Python would require a new workflow using the functions contained in *decoration_manager.py*. While these tools represent a significant progress in automated rapid generation and modification of 3D molecular geometries, we sought to develop an easy-to-use tool which can quickly create reasonably accurate molecular geometries in the local chemical space of a given molecular scaffold.

**Fig. 1:**
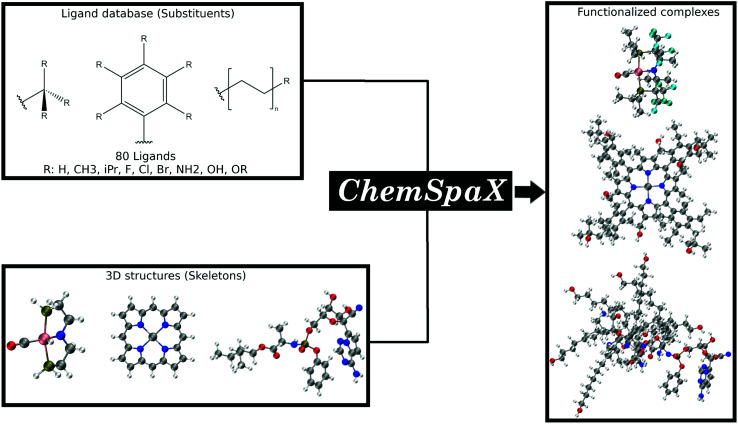
A general overview of the approach used in *ChemSpaX*. The local chemical space of a pre-optimized input molecular scaffold can be explored by automatically placing ligands from a pre-defined ligand library. Color code used for elements: gray = C, white = H, red = O, pink = Ru, dark-blue = N and turquoise = F.

In this manuscript we present *ChemSpaX*, a Python-based workflow that can be used for automated exploration of the chemical space of molecules. The exploration is done by automated placement of substituents on a given molecular scaffold while maintaining the quality of the initial scaffold. If a particular complex is known to be catalytically active, the 3D coordinates of this complex can be used as a starting point *via ChemSpaX* for exploration in the neighbourhood of its chemical space. The user has full control of the placement of substituent groups and can guide the exploration of the local chemical space. A general overview of the approach used in *ChemSpaX* is given in Fig. 1.

The computational methods employed in this manuscript are presented in the next section. A description of the code implementation to develop *ChemSpaX* follows. Subsequently, representative applications of *ChemSpaX* are presented. First, we applied *ChemSpaX* to generate a database of ∼1100 functionalized Cobalt Porphyrin (referred to as ‘Co porphyrin’ in the rest of this manuscript) complexes. Co porphyrins exist as stable metalloradicals and are used to catalyze carbene and nitrene transfer reactions.^57–60^ It has been shown that the quality of geometries obtained at the GFN2-xTB level of optimization are reasonably accurate compared to DFT.^61–66^ We therefore use the generated database to investigate if the quality of geometries generated by *ChemSpaX* is reasonably close to GFN2-xTB optimized geometries and to investigate the propagation of errors‡‡Propagation of errors in this context means that complexes that are getting increasingly more complex are being compared to their DFT optimized ‘standard’. upon creation of larger geometries. Additionally, a comparison of HOMO–LUMO gaps calculated by GFN2-xTB and DFT is made.

Next representative applications involve Ru and Mn catalysts based on pincer ligands. Pincer ligands have emerged as versatile ligand platforms enabling a plethora of catalytic reactions.^67–70^ The functionalization of a RuPNP pincer complex derived from the commercially available Ru-MACHO is described.^28^ TMCs based on the MACHO ligand framework have shown versatile activity in catalyzed (de)hydrogenation reactions.^71–76^ Analyzing the chemical space of the Ru-MACHO catalytic scaffold can be a valuable asset for multiple applications. Next, the functionalization of Mn–pincer complexes as potential (de)hydrogenation catalysts is studied.^61^ With this application the chemical space of an earth-abundant alternative to RuPNP is explored. Manganese is known to be a cheap, abundant and biocompatible alternative to precious-metal catalysts.^73,77–79^ The quality of geometries generated by *ChemSpaX* is compared against higher-level DFT and GFN2-xTB methods.

Finally, the functionalization of a bipyridyl functionalized cobalt-porphyrin trapped in a M_2_L_4_ type cage complex (referred to as ‘M_2_L_4_ cage’ in the rest of this manuscript) is presented. This cage complex confines a Co porphyrin complex, which can lead to changed catalytic properties.^60,80,81^ The M_2_L_4_ cage is a challenging and cumbersome scaffold to functionalize manually. The quality of *ChemSpaX* generated M_2_L_4_ cage geometries is compared against semi-empirical and force-field based xTB optimization methods. This case shows how *ChemSpaX* can be used to functionalize diverse and challenging molecular scaffolds. We note here that while all our representative examples are TMC focused, *ChemSpaX* can be applied to other molecular scaffolds that do not necessarily contain a TM center as well.

## 2 Computational methods

### 2.1 Open Babel

Conversions between MDL Molfile and *XYZ* format were done using Open Babel.^82,83^ Open Babel was also used to perform Generalized Amber Force Field (GAFF), and the Universal Force Field (UFF) optimizations.^84,85^

### 2.2 Semiempirical tight-binding

Grimme lab's xTB package (version 6.3.3) was used for semi-empirical tight-binding calculations.^86^ The GFN2-xTB, and GFN-FF methods were used for geometry optimization.^87–90^ The GFN*n*-xTB methods are quantum chemistry based semiempirical methods which extend the original density functional tight binding model. GFN-FF is a nonelectronic, force-field version of the GFN approach. The M_2_L_4_ cage geometries were optimized using GFN2-xTB and GFN-FF. The GBSA solvation method as implemented in xTB was used with THF as solvent for most optimizations to implicitly account for solvent effects.^91,92^ Thermochemical parameters such as the Gibbs free energy were computed using the hessian matrix calculations. These GFN*n* (*n* = 2, FF) methods are denoted as GFN*n*-xTB(THF) or GFN*n*-xTB(GAS) depending on whether GBSA solvation was used.

### 2.3 Density functional theory

#### 2.3.1 Pincer complexes

Gaussian 16 C.01 was used to perform DFT calculations.^93^ The BP86 exchange–correlation functional was used for geometry optimizations together with the def2SVP basis set.^94,95^ This combination of functional and basis set have shown reliable geometry predictions accompanied with low computational costs.^96,97^ Geometry optimizations were performed in the gas phase. Hessian calculations were performed for these geometries to verify the absence of imaginary frequencies and that each geometry corresponded to a local minimum on its respective potential energy surface (PES). Thermochemical parameters such as the Gibbs free energy were computed using the gas phase hessian calculations. Single point (SP) DFT calculations were performed on the gas-phase optimized geometries using the SMD solvation (THF) model.^98^ SP calculations were performed using BP86 or PBE1PBE (also known as PBE0) functional with the def2TZVP basis set to further refine the obtained (free) energies and other thermochemical/electronic properties.^95,99^ All DFT calculations were performed with Grimme's D3 dispersion corrections.^100^ These composite methods (geometry optimization followed by SP), BP86/def2-SVP//XC/def2-TZVP (THF), are denoted as XC(THF) or XC(GAS) depending on the exchange–correlation (XC) functional used. All geometries were pre-optimized with a combination of Openbabel's GAFF and UFF methods and/or GFN2-xTB before being subjected to full DFT based optimization. No conformational search was conducted after or during geometry optimizations.

The catalysts are denoted as M-L where M represents the metal center and L the ligand. Reactive adsorption of a H-X species (X = H, Br, OH, i-PrO) over M-L leads to the formation of M(X)-L(H) species. The thermodynamic stability of the M(X)-L(H) was estimated by computing the Gibbs free energy and total energy change under standard conditions upon addition of the H-X moiety.

1 H-X + M-L → M(X)-L(H)

2
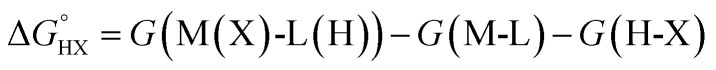
3
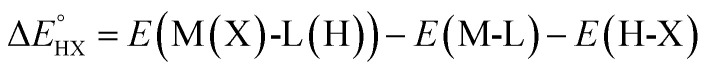


#### 2.3.2 Co porphyrins

TeraChem v1.94V-2019.08-beta was used to perform GPU-accelerated DFT SP calculations using the PBE1PBE XC and LANL2DZ basis sets with an effective core potential (ECP) on selected Co-porphyrin geometries optimized using the GFN2-xTB method.^101–104^

### 2.4 Root-mean-square deviation of atomic positions (RMSD)

The RMSD is used to compare two molecular structures. In this approach, the minimal difference between the positions of the same atom on both molecular structures is used. The cartesian heavy-atom (all elements except H) root-mean-square deviation (hRMSD) is an often used metric to compare molecular geometries produced from different methods.^86^ The RMSDs and hRMSDs were calculated using a Python program which uses the Kabsch or Quaternion algorithm to align the two molecular structures and calculate the minimum (h)RMSD over all possible alignments.^105–107^ If for example the two molecules p and q with n points (atoms) are compared, the RMSD is defined as:4
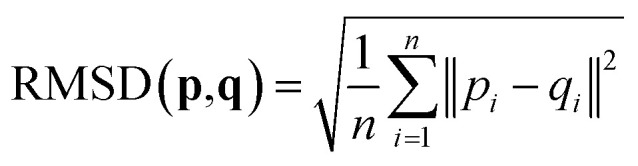
5
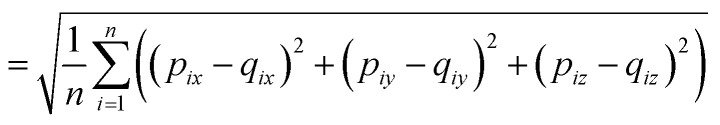


### 2.5 Machine learning methods

For the Mn–pincers, automated ML using the TPOT library in Python was applied to perform ML assisted HOMO–LUMO gap prediction.^108–110^ TPOT allows for optimization of machine learning pipelines using genetic programming. The molecular structure of each functionalized complex was represented as a Coulomb matrix generated using the qmlcode package.^111^ We used the *generate_coulomb_matrix* functionality of the qmlcode package with parameters *size = 200* and *sorting = “row-norm”*. We used the GPU version of TPOT to search for a ML model using the coulomb matrix representation of the FF geometries. The ML pipeline from the first generation of TPOT run resulted in an XGBRegressor based ML model. The model with the related hyperparameters is provided in the ESI (see S12).† XGBRegressor uses the extreme gradient boosting algorithm which includes an ensemble of ML algorithms constructed *via* decision tree models. This ML model was used to learn the HOMO–LUMO gap for both FF and DFT geometries using the same hyperparameters and using the Coulomb matrix representation as a descriptor. The Pearson correlation coefficient (*R*^2^_score) was used as a metric for the optimization of the ML model. The dataset was split into the training and test sets (75–25 split). The ML model was trained on the training set and its performance was tested on a test set, which it had not seen before.

## 3 Code implementation

**Fig. 2:**
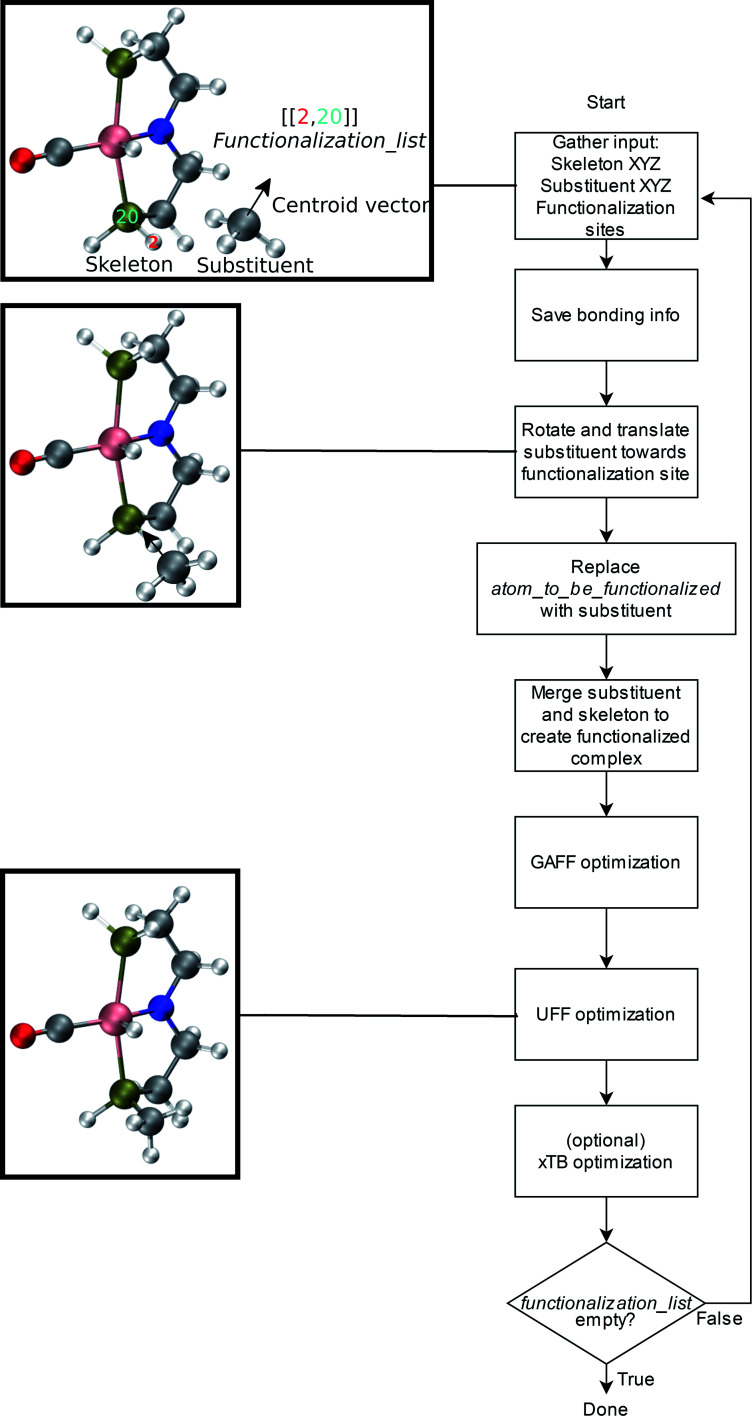
Overall workflow of ChemSpaX. (1) The user supplies the *XYZ* coordinates or MDL Molfile of a molecular skeleton, *functionalization_list*, and substituents. The *atom_to_be_functionalized* (index 2) and *bonded_atom* (index 20) are indicated in the skeleton's XYZ or MDL Molfile. (2) If *XYZ* files are supplied, they are converted to MDL Molfiles using Open Babel.^113^ If MDL Molfiles are already provided, they are used to conserve correct bonding info. This bonding info is used in step 5. (3) The central atom of the substituent group and the centroid vector are used to rotate and translate the substituent group towards the functionalization site. (4) *atom_to_be_functionalized* is replaced by the substituent group. (5) The skeleton and substituent group are merged in one MDL Molfile with correct bonding information from input MDL Molfiles. (6) GAFF optimization is done to prevent steric hindrance. (7) Additionally, UFF optimization is done to prevent GAFF related issues. (8) Optionally, xTB optimization can be used for further optimization of the full functionalized skeleton. (9) If there are no functionalizations left to do, the program exits and the functionalized skeleton is saved in MDL Molfile format. Else the functionalized skeleton will be used as input and the entire process repeats from step 1.

An overview of the workflow of ChemSpaX is shown in Fig. 2. The user has to supply: a molecule that needs to be functionalized (*skeleton*), which sites on the skeleton should be functionalized (*functionalization_list*) and what substituent should be placed on the supplied site (*substituent*). The *functionalization_list* can contain several functionalizations. Each functionalization is an ordered pair of indices [*b*, *a*]. Here, *a* is the index of the atom bonded to the scaffold, and *b* corresponds to the atom which is replaced upon functionalization. This corresponds to indices *b* = 2 and *a* = 20 in Fig. 2. Substituents can be chosen from a pre-made database shipped with *ChemSpaX* or users can create new substituents in *XYZ* or MDL Molfile format.^112^ Information for the correct placement of a substituent is kept in a *CSV* file. The *CSV* file stores: (1) coordinates of the *central atom* of the substituent group which coordinates directly to the sites defined in the *functionalization_list*. (2) Coordinates of the *centroid vector*. A centroid vector, for a tetrahedral substituent such as CH_3_ is the vector connecting the central atom (C) to the centroid of the triangle formed by the three edges (hydrogens). For a planar substituent such as NH_2_ the centroid vector connects the center of the line joining the two hydrogen atoms to N (central atom).

The substituent group is first correctly oriented using rotation matrices and placed at a pre-defined distance from the substitution site. This process is described in more detail in the ESI.† Next, GAFF followed by UFF optimization methods from Open Babel were used to selectively optimize the newly placed substituent *via* a constrained optimization protocol while keeping the original molecular skeleton frozen.^82,83^ This combination of GAFF and UFF was found by trial-and-error (see ESI†). The correction in orientation of the substituent group prior to FF optimizations is needed to ensure a reasonable input geometry. The FF optimizations are based on MDL molfiles which contain the connectivity information between different atoms. When new substituents are placed the connectivity information is carefully updated to ensure that only desired connections are present. FF optimization using the MDL molfiles as input rectifies the geometry and removes any physical overlap between different atoms/functional groups. Optionally, an xTB optimization of the whole functionalized skeleton (including the new substituent) can be done.

It is recommended to use a DFT optimized geometry as input skeleton since the FF optimization only influences the newly placed functional group. This choice helps keep the core of the geometry as close to its DFT optimized input structure as possible upon serial functionalization, while preventing steric hindrance from newly placed substituents cheaply *via* force-field optimizations. *ChemSpaX* uses FF optimizations on newly placed substituent groups in each iteration. Therefore we name geometries generated directly by *ChemSpaX* as “FF geometries”.

## 4 Results and discussion

### 4.1 Co-porphyrin

Porphyrins are widely investigated, for example, for their applications in biocatalysis, organic photovoltaics, molecular wires and many more applications.^2,114–116^ This wide variety of applications has been enabled by the design and synthesis of structurally diverse porphyrins.^2^ PF has been widely used to tune the electronic and chemical properties of porphyrins, where functional groups and substituents are introduced after the construction of the porphyrin macrocycle. However, experimental exploration of the chemical space of porphyrins is limited by synthetic and economic feasibility. This is where computer-aided molecular design tools can be helpful.

Apart from the chemical application perspective, investigating the functionalization of porphyrins is also of use for further refinement of our workflow. When functionalizing a structure as implemented in *ChemSpaX* (freezing the skeleton and performing FF optimization only on the newly placed substituents), errors can be introduced. Stretching or compression of the skeleton structure is not taken into account since the skeleton is frozen. By investigating a structure that is close to 2D instead of 3D, like porphyrins, the assessment of the introduced errors and their propagation through the workflow of *ChemSpaX* is simplified.

We performed functionalization of Co porphyrins following a serial functionalization strategy§§A serial functionalization strategy means that the functional groups were placed one after another, leading to functionalized structures that are increasingly getting more complex. to create a database of ∼1100 complexes. Section 4.1.1 discusses the functionalization strategy. Subsequently, the propagation of errors introduced in geometries generated by *ChemSpaX* is investigated in Section 4.1.2.

#### 4.1.1 Functionalization strategy

**Fig. 3:**
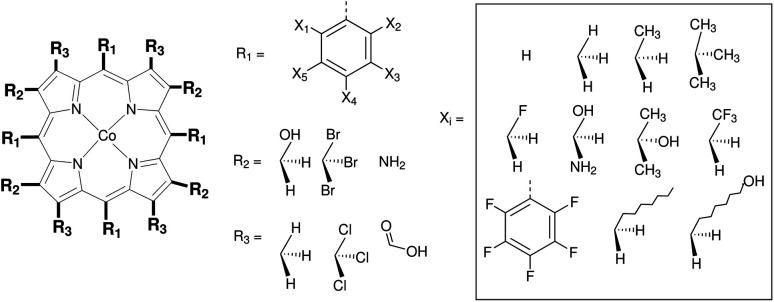
Functionalization strategy for Co porphyrin. Phenyl groups were first placed on the R_1_ sites and subject to further functionalizations. With this strategy a database of 1120 Co porphyrin structures was generated.

Fig. 3 shows the functionalization strategy for Co porphyrin. The Co porphyrin skeletons were functionalized with various phenyl groups on the R_1_ sites to generate 10 new skeletons. These skeletons were then functionalized on 28 sites on the R_2_ and R_3_ sites. This was done with 4 sets of 28 substituents. This workflow generated 1120 functionalized Co porphyrin complexes (10 × 28 × 4 = 1120). The functionalization was done serially as described in Code implementation. The sites X_1_–X_5_ on the phenyl rings (R_1_) were functionalized first. Finally, functionalizations were done on R_2_ and R_3_ respectively.

**Fig. 4:**
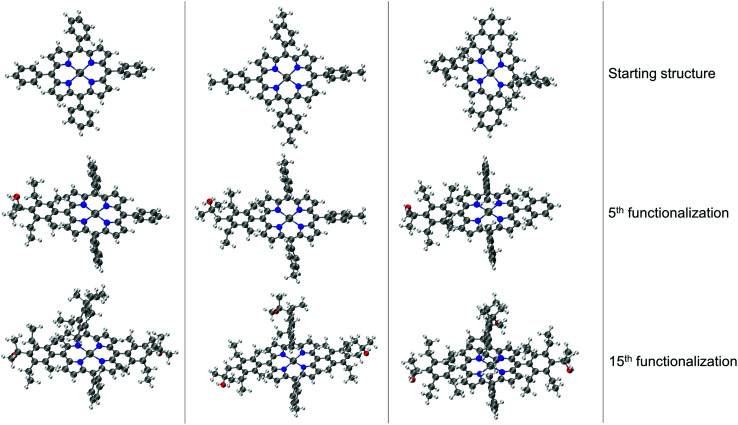
Functionalization strategy for Co porphyrin shown for 3 different skeletons. For each skeleton geometries resulting from the 5^th^, and the 15^th^ functionalization are shown in a column. The phenyl rings are functionalized symmetrically. In the 5^th^ functionalization the left most phenyl ring of the skeleton is functionalized, in the 10^th^ functionalization step the same substituents are placed on the upper phenyl ring, and on the right most phenyl ring in the 15^th^ step. Color code used for elements: gray = C (metal center = Co), white = H, red = O, dark-blue = N and turquoise = F.

The resulting complexes are shown for 3 different skeletons in Fig. 4, where the skeleton, and Co-porphyrin complexes at the end of 5^th^, and 15^th^ functionalization are shown in a column demonstrating the geometric complexity introduced upon functionalization.

#### 4.1.2 Error propagation of serial functionalization

**Fig. 5:**
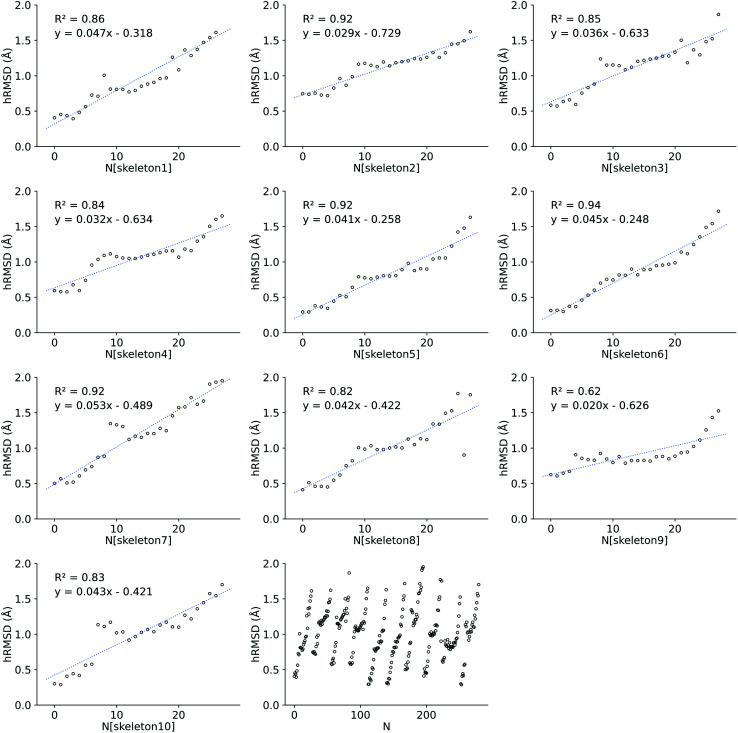
Increasing hRMSD for each functionalization on a given skeleton. Where *N* is the number of functionalizations, starting from 0. 10 skeletons were created and 28 functionalizations were done for each skeleton. The first 10 blocks each represent a skeleton, while the last block on the bottom shows the increasing hRMSD for each skeleton grouped in 1 figure. After every 28^th^ functionalization (0 ≤ *N* ≤ 279), a new skeleton is functionalized.

The 1120 geometries were optimized using GFN2-xTB(THF). The hRMSD between FF and GFN2-xTB optimized geometries was computed to compare the quality of the FF geometries generated by *ChemSpaX*, giving *μ*_*h*RMSD_ = 1.28 ± 0.54 Å. Upon detailed analysis of the hRMSD it was observed that the hRMSD increases nearly linearly for each subsequent functionalization on a skeleton. The error introduced by placing a new substituent group is thus propagated upon the next placement of a substituent. An example is shown in Fig. 5 where the hRMSD for each skeleton is plotted. The first 10 blocks of plots show the increasing hRMSD for each functionalization on a given skeleton. The last block shows the hRMSDs for all 10 skeletons, showing how the error increases almost linearly upon each functionalization regardless of the skeleton used for functionalization. At the start of each functionalization run, the error is minimal since a DFT optimized skeleton is used. It might thus be important to use intermediate optimizations with a higher level of theory during a large serial functionalization run. These results could help users in devising an optimal strategy to get more accurate geometries at a balanced computational cost when using *ChemSpaX*. One can determine when an extra geometry optimization with a higher-level method, such as GFN2-xTB, is needed in-between functionalizations. The linear regression fits shown in Fig. 5 can be used to estimate the hRMSD. One can generate these relations on a small sub-set of geometries in the functionalization scope by performing additional geometry optimizations at a higher level of theory. The predicted hRMSD can be used to set a threshold value. When this threshold is reached, a higher-level optimization method can be used to reduce the hRMSD and the functionalization can be continued with the optimized intermediate as a new skeleton.

We also investigated the HOMO–LUMO gap of Co porphyrins within a serial functionalization run. DFT SP calculations on 28 GFN2-xTB optimized geometries and FF optimized geometries were done to calculate the HOMO–LUMO gap. These 28 geometries were obtained in a single serial functionalization run. The HOMO–LUMO gaps of GFN2-xTB optimized geometries and FF optimized geometries (see ESI†) showed a reasonable linear correlation (*R*^2^ = 0.68 RMSE = 0.10 eV). Consequently, in contrast to hRMSD, the difference in computed HOMO–LUMO gaps (GFN2-xTB_HL-gap_ − FF_HL-gap_), did not show a growing trend within the functionalization run.

**Fig. 6:**
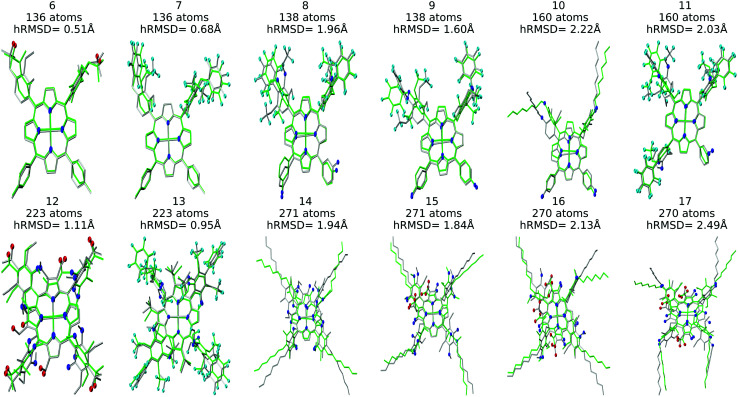
Structure overlay plots of selected Co porphyrin complexes. ChemSpaX generated (FF) structures (silver) are plotted against GFN2-xTB optimized (silver) structures. The upper half of the figure consists of structures with <200 atoms and the lower half of the figure shows structures >200 atoms. Color code used for elements: red = O, dark-blue = N and turquoise = F.

Structure overlay plots of the FF geometries (silver) and the GFN2-xTB optimized geometries (green) are shown in Fig. 6. The upper half of the figure shows structure overlay plots of complexes with <200 atoms while the lower half shows complexes with >200 atoms, and diverse hRMSDs. Large hRMSDs result from divergent orientation of long alkyl chain substituents, and fluorine containing groups.

### 4.2 Pincer complexes

In this section the functionalization of the ligand scaffold of Ru and Mn based pincer complexes is described. In Section 4.2.1 the functionalization strategy for both types of pincer complexes is shown. Subsequently, the quality of geometries generated by *ChemSpaX* is compared to higher level methods in Section 4.2.2. Prediction of DFT computed HOMO–LUMO gap using a machine learning approach is presented in Section 4.2.3.

#### 4.2.1 Functionalization strategy

**Fig. 7:**
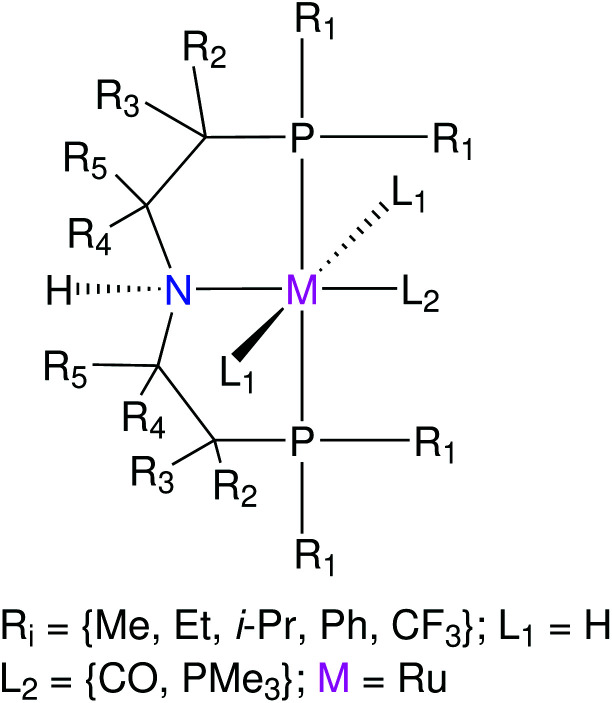
Functionalization strategy for the RuPNP pincer complexes.

The functionalization strategy for the RuPNP complex is shown in Fig. 7. This functionalization strategy resulted in 144 (M-L) complexes. For each M-L complex, its hydrogenated version M(H)-L(H) was also generated by functionalization of the M(H)-L(H) complex as skeleton, leading to a total of 288 geometries. Out of these, 27 pairs (M-L/M(H)-L(H)) were selected for BP86(GAS) optimization. BP86(THF) and PBE1PBE(THF) SP calculations were performed on the DFT optimized geometries.

Krieger and co-workers^61^ used an “*under development*” version of *ChemSpaX* to generate a database of 1225 Mn complexes based on five representative ligand scaffolds namely PNP-(bis(3-phosphaneylpropyl)amine)-¶¶This is not the same PNP ligand as used for the Ru complexes. The PNP ligand used for Mn complexes contains a propyl bridge while the one used for Ru complexes contains an ethyl bridge., SNS (azanediylbis(ethane-1-thiol)), CNC (bis(2-(1H-3λ^4^-imidazol-3-yl)ethyl)amine), PNN (N^1^-(2-phosphaneylethyl)ethane-1,2-diamine), and PCP (N^1^,N^3^-bis(phosphaneyl)benzene-1,3-diamine).^117–123^ Out of these 1225 geometries, we chose 365 geometries containing the PCP and CNC ligand backbones for full DFT based optimization using BP86(GAS). This dataset of 365 complexes will be discussed further.

**Fig. 8:**
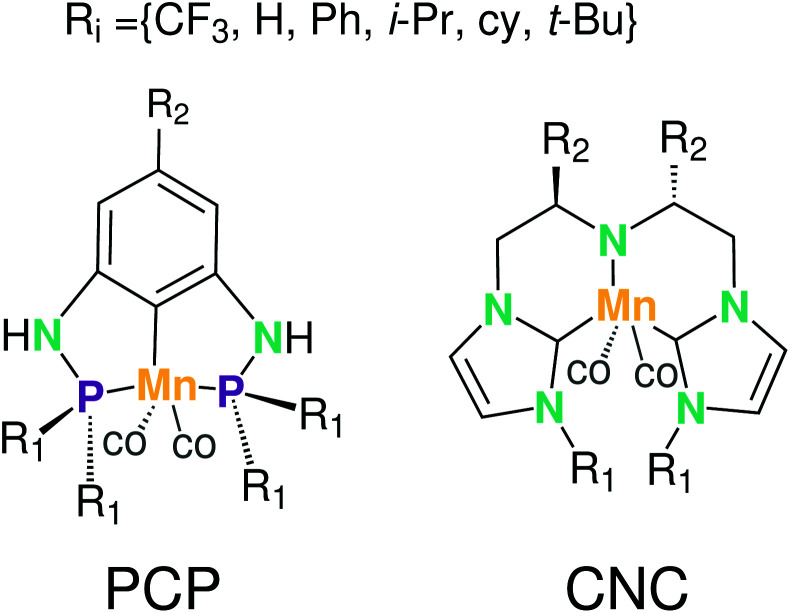
Functionalization strategy for Mn–pincers with various donor (R_1_) and backbone (R_2_) groups.

The functionalization strategy for PCP and CNC ligand based complexes is shown in Fig. 8. Functionalizations were performed symmetrically keeping all four R_1_ sites the same. Similarly both R_2_ sites (only 1 in case of PCP backbone) were functionalized with the same groups. However, R_1_ and R_2_ were not constrained to be the same.

#### 4.2.2 Quality assessment of generated geometries

To assess the quality of FF geometries, they were compared against GFN2-xTB and DFT. The quality of geometries was assessed along two dimensions: energy and spatial. When comparing geometries along the *energy* dimension, we computed the net energy change of a particular chemical reaction using SP calculations on FF geometries at higher levels of theory (*e.g.* DFT or GFN2-xTB). For a given reaction the *ChemSpaX* generated FF geometries of reactants and product were taken. Each FF geometry underwent two calculations: (1) a SP calculation where the energy of the FF geometry was evaluated at the DFT level of theory and the electronic energy change of the reaction Δ*E*_FF//DFT−SP_ was calculated (2) a full DFT based optimization was carried using the FF geometry as input resulting in a new geometry and energy at the DFT level of theory. The corresponding electronic energy change of the reaction Δ*E*_DFT_ was calculated. The difference between the reaction energies is computed using Δ*E*_FF//DFT-SP_ and Δ*E*_DFT_ to get ΔΔ*E*_FF_ which is a metric for the quality of FF geometry for the reaction being investigated (eqn (6)). A similar approach was used by Sinha and co-workers to assess the quality of GFN2-xTB optimized geometries against DFT (eqn (7)).^62^

6 ΔΔ*E*_FF_ = Δ*E*_DFT_ − Δ*E*_FF//DFT-SP_

7 ΔΔ*E*_GFN2-xTB_ = Δ*E*_DFT_ − Δ*E*_GFN2-xTB//DFT-SP_

Such a comparison allows us to estimate the range of error caused by the direct use of FF geometries by skipping computationally expensive DFT optimizations, for example in high-throughput screening (HTS) workflows. Geometries were also assessed along the *spatial* dimension where we computed the RMSD between FF-geometries against GFN2-xTB, and DFT optimized geometries.

For RuPNP complexes we chose the hydrogenation reaction (M-L + H_2_ → M(H)-L(H)) to investigate ΔΔ*E*_FF_ and ΔΔ*E*_GFN2-xTB_. The corresponding mean (*μ*) and standard deviation (*σ*^2^) were also computed.||||ΔΔ*E*_GFN2−xTB_: 20 pairs; (ΔΔ*E*_*FF*_) 25 pairs M–L/M(H)–L(H) geometries. We found *μ*(ΔΔ*E*_FF_) = 7.20 ± 4.57 kcal mol^−1^; and *μ*(ΔΔ*E*_GFN2-xTB_) = 4.77 ± 2.57 kcal mol^−1^. This indicates a qualitatively good agreement between the GFN2-xTB optimized structures and the structures generated by *ChemSpaX*.

For the Mn–pincer complexes we investigated reactive adsorption of H-X species (M-L + H-X → M(X)-L(H); X = H, Br, i-PrO, and OH) with the reaction energetics characterized by Δ*E*, and the respective FF geometries by ΔΔ*E*_FF_ respectively. FF geometries of Mn–PCP complexes were found to have the lowest ΔΔ*E*_FF_ in general, followed by the Mn–CNC complexes. For the formation of M(X)-L(H) adducts, ΔΔ*E*_FF_ were found to be lowest for the reactive adsorption of HBr and H_2_, and highest for i-PrOH and H_2_O. The reason for worse performance of i-PrOH and H_2_O can be attributed to the observed recombination of O–H bond in many geometries during DFT based optimization leading to M(H-X)-L type complexes, or non-adsorbed H-X adducts. We further observed that ΔΔ*E*_FF_(HBr) and ΔΔ*E*_FF_(H_2_) correlated well (Pearson correlation coefficient (*R*) = +0.87) opening up possibilities to reduce computational effort in screening through these intermediates. Krieger and co-workers had reported a similar albeit stronger correlation (*R* = 0.95) between Δ*G*_HBr_ and Δ*G*_H2_.^61^ A detailed discussion of the effect of functionalization of the ligand scaffold on the ΔΔ*E*_FF_ values is presented in the ESI.†

**Fig. 9:**
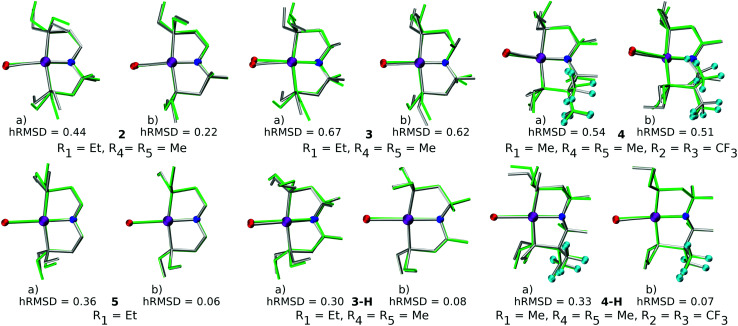
Structure overlay plots of some selected RuPNP complexes. (a) FF optimized (silver) *vs.* DFT optimized (green) structures and (b) GFN2-xTB optimized (silver) *vs.* DFT optimized (green) structures. The ‘**–H**’ indicates that the complex is hydrogenated. Color code used for elements: red = O, purple = Ru, dark-blue = N and turquoise = F.

The quality of FF geometries along the spatial dimension was analyzed *via* hRMSD between the geometries produced by DFT or GFN2-xTB based optimizations.^86^ For the RuPNP complexes, both FF (0.67 ± 0.30 Å) and GFN2-xTB (0.41 ± 0.34 Å) structures had a similar average hRMSD when compared to DFT structures. A selection of the RuPNP geometries are visualized using structure overlay plots in Fig. 9. The comparisons in these structure overlay plots is done as follows: (a) the FF optimized structure (silver), generated by *ChemSpaX*, is compared to a DFT optimized structure (green) and (b) a GFN2-xTB optimized structure (silver) is compared to a DFT optimized structure (green).

**Fig. 10:**
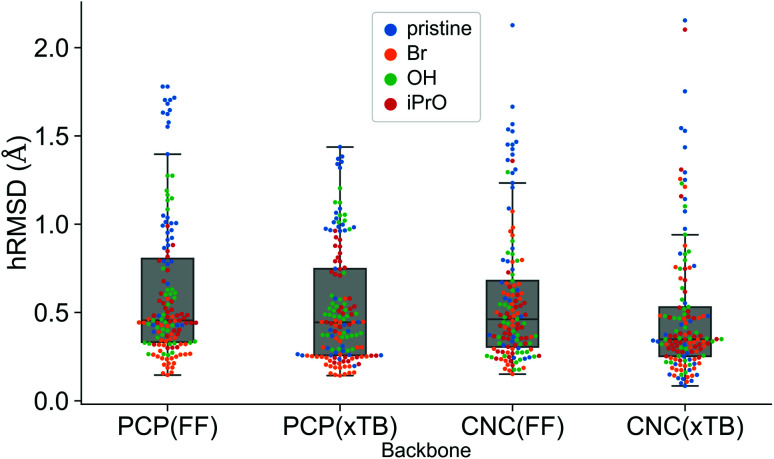
Comparison of hRMSD for the PCP and CNC ligand backbone of *ChemSpaX* generated FF structures, and GFN2-xTB optimized structures compared to DFT optimized structures. The various adducts bonded to the metal center are color coded, where ‘pristine’ means that no adduct is bonded to the metal center.

A comparison using the hRMSD was done in a similar manner for the Mn–pincer complexes. It was again observed that both FF (0.60 ± 0.40 Å) and GFN2-xTB (0.51 ± 0.36 Å) structures had a similar average hRMSD when compared to DFT structures, albeit with moderately high standard deviations. It should be noted here that the GFN2-xTB optimizations were performed in the solvated phase (GBSA(THF)) while the DFT optimizations are in the gas phase. The hRMSD analysis was performed for PCP and CNC ligand backbones (see Fig. 10). For the CNC backbones it was observed that functionalization with electron donating substituents on the R_1_ site resulted in a higher hRMSD. For the PCP backbone it was observed that functionalization with *t*-Bu on the R_1_ site specifically gave a larger hRMSD. This observation is expected to have the following underlying causes: (1) electron donating groups like *t*-Bu are bulkier, have more number of atoms and a complex structure which increases the chance of accumulating an error (*vide infra*) (2) electron donating groups affect the electronic density in the entire complex and may elicit shearing of the skeleton which is kept frozen in FF calculations. When optimized with DFT, the skeleton would relax and this would lead to a higher hRMSD.

We would like to note here that all the FF geometries discussed above were generated using a serial functionalization scheme. For each complex a skeleton was chosen, functionalization sites were defined, and functional groups were placed on the skeleton one after another without any intermediate optimizations at a higher level of theory. Since the skeleton is not relaxed it is likely to accumulate errors (higher hRMSD; higher ΔΔ*E*_FF_) as the size complexity increases. The hRMSD and ΔΔ*E*_FF_ values would be lower if intermediate optimizations are performed (*vide supra*).

#### 4.2.3 ML assisted prediction of HOMO–LUMO gap

HOMO–LUMO gap prediction based on only the molecular structure as input using statistical methods can be a great resource to screen and develop functional inorganic materials.^124^ As a proof of concept, a machine learning model using the XGBoost regressor (see ESI†) as available *via* the TPOT library in Python was applied to perform ML assisted HOMO–LUMO gap prediction.^125^

**Fig. 11:**
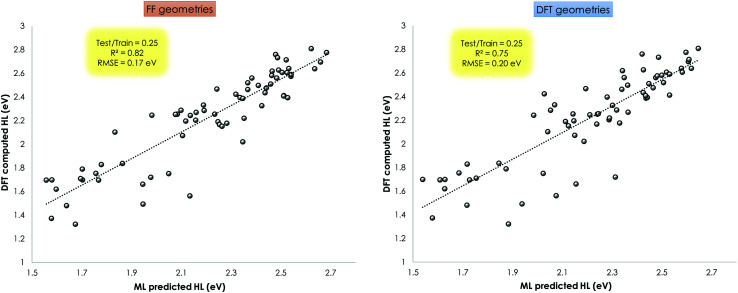
Prediction of DFT (BP86(THF)) computed HOMO–LUMO gap using Coulomb matrix representation of geometries produced by ChemSpaX (FF geometries; left), and DFT optimized geometries (right). These figures show the *R*^2^ and RMSE of the model fitted to the test set.

This ML assisted prediction of the HOMO–LUMO gap was done for the Mn–pincers with PCP and CNC ligands. The results are shown in Fig. 11. It is shown that for this small dataset, the *ChemSpaX* generated FF geometries can already give reasonable predictive power to a ML model (*R*^2^ = 0.82 RMSE = 0.17 eV) which is close to results obtained using DFT optimized geometries (*R*^2^ = 0.75 RMSE = 0.20 eV). It is noteworthy that the HOMO–LUMO gap computed on FF geometries weakly correlate with the HOMO–LUMO gap computed on DFT optimized geometries (*R*^2^ = 0.36; see ESI†). The linear regression fit results in a rather high RMSE of 0.6 eV to predict the HOMO–LUMO gap of DFT optimized geometries using the HOMO–LUMO gap computed for FF geometries. The ML model which uses the FF geometries as input, avoids additional DFT calculations, and provides a reasonably accurate prediction of HOMO–LUMO gaps, therefore directly reflects the usefulness of FF geometries (Fig. 11).

### 4.3 M_2_L_4_ cage

**Fig. 12:**
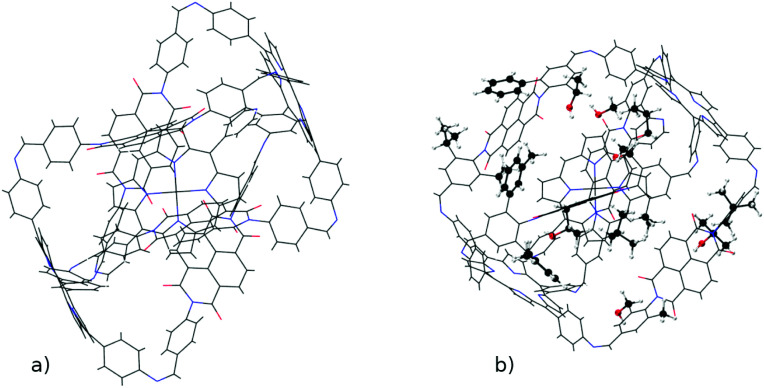
A visualization of the functionalized M_2_L_4_ cage which shows (a) the input skeleton and (b) the GFN2-xTB optimized geometry after placement of 16 substituents. The newly placed substituents are shown in a distinguished representation.

The versatility of *ChemSpaX* is shown by the automated placement of substituents without introducing steric hindrance on a geometry that is more complex. An M_2_L_4_ cage was functionalized at 16 sites with various substituent groups. The results are shown in Fig. 12. This serial functionalization yielded 16 structures and these were further optimized using GFN-FF and GFN2-xTB(GAS). The hRMSDs between FF geometries and those generated using the GFN-FF and GFN2-xTB optimization methods were calculated. The statistics shown for each optimization method in Table 1 revealed that the *ChemSpaX* generated FF geometries are closer to the GFN2-xTB optimized geometries compared to GFN-FF optimized geometries.

**Table 1:** Statistics for the hRMSD between various methods. The two optimization methods that are compared to each other are shown in the first row

	GFN-FF *vs.* GFN2-xTB	FF *vs.* GFN2-xTB	FF *vs.* GFN-FF
*μ*	2.54 Å	2.14 Å	0.83 Å
*σ* ^2^	0.34 Å	0.25 Å	0.26 Å
Max. hRMSD	3.18 Å	2.46 Å	1.37 Å

### 4.4 Remarks regarding *ChemSpaX*

**Fig. 13:**
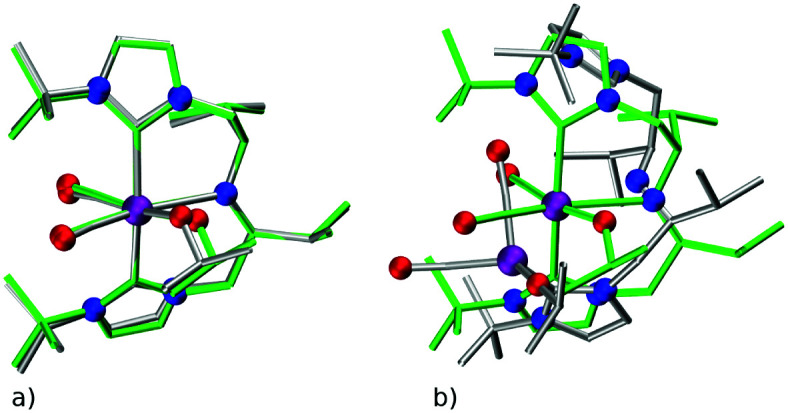
Structure overlay plots of selected hemi-labile Mn pincers with a PNN backbone. (a) FF optimized (silver) *vs.* a DFT optimized structure (green) and (b) GFN2-xTB optimized (silver) *vs.* a DFT optimized structure (green). Color code used for elements: red = O, purple = Mn and dark-blue = N.

There are two key points of discussion related to the current work. First, it should be noted that (at least some of the) *ChemSpaX* generated geometries should be checked during serial functionalization. Such a check could be done by manual inspection of randomly selected geometries, or could be performed *via* descriptors such as a RMSD of the skeleton, and selected bond length and angle. Such checks are crucial since functionalization of the skeleton can significantly alter the geometry in some cases, for example, *via* ligand hemi-lability. In an early application of *ChemSpaX*, Krieger and co-workers found that some of the Mn–pincer complexes with a PNN backbone became hemi-labile upon functionalization.^61^ Such hemilability was only discovered after GFN2-xTB/DFT based geometry optimizations were performed on *ChemSpaX* generated geometries. This resulted in high hRMSDs and high ΔΔ*E*_FF_. Note that Mn–PNN complexes also proved challenging for GFN2-xTB calculations and high hRSMDs and ΔΔ*E*_GFN2-xTB_ values were found. Structure overlay plots of a selected case of Mn–PNN demonstrating the challenges associated with hemi-labile PNN ligand are shown in Fig. 13. The comparison is again done as follows: (a) the FF optimized structure (silver), generated by *ChemSpaX* is compared to a DFT optimized structure (green) and (b) a GFN2-xTB optimized structure (silver) is compared to a DFT optimized structure (green). These complexes, before being subject to DFT based geometry optimizations, were pre-optimized using GFN2-xTB which yielded hemi-labile geometries, and biased the DFT optimizations to also converge to hemi-labile structures. Despite larger hRMSD values, it is noteworthy that majority of FF geometries still have hRMSD < 1 Å, and are overall lower than hRMSD values for the GFN2-xTB geometries (see ESI†).

Another noteworthy point is that the purpose of *ChemSpaX* is to explore the local chemical space by placing functional groups on a given molecular scaffold. *ChemSpaX* is not designed for conformational search which can be important for exploration of the internal chemical space of a molecule. We recommend users to employ other software based solutions such as xTB's CREST simulations for this purpose.^126–128^

## 5 Summary and conclusions

In this work, an automated Python-based workflow for the exploration of local chemical space is presented. *ChemSpaX* can place substituents on specific sites of diverse molecular scaffolds based on initial user input and uses FF optimization to optimize newly placed substituents. Use cases were shown by using a data-augmented approach which utilized fast GFN2-xTB optimizations to compare structures generated by *ChemSpaX*. For selected use cases a comparison was also done against DFT optimized structures. Descriptors such as the HOMO–LUMO gap that can be used for HTS applications were studied in more detail for some of the presented use cases. Analysis of functionalized Co porphyrins generated by *ChemSpaX* showed that the hRMSD increased nearly linearly with the number of atoms in serial functionalization runs on a given skeleton. This observation paves the way for devising a strategy to optimally employ higher-level geometry optimizations in intermediate steps. For the pincer complexes the quality of geometries generated by *ChemSpaX* was assessed along the energy (ΔΔ*E*) and spatial (hRMSD) dimensions. Generally FF geometries were found to be similar in quality compared to GFN2-xTB optimized geometries based on the hRMSDs, and ΔΔ*E*. It was discovered that *ChemSpaX* generated FF geometries can be used to train a ML model which can predict the DFT calculated HOMO–LUMO gap with reasonable accuracy, which can be useful in accelerated property prediction and catalyst screening. Additionally, it has been demonstrated that diverse molecular scaffolds can be functionalized using *ChemSpaX*.

To conclude, *ChemSpaX* can be used to generate satisfactory 3D geometric representations in the local chemical space of a given molecular scaffold, particularly including TM complexes. The generated structures, in conjunction with quantum chemical and statistical methods, can be used to generate structure–property databases enabling data-driven chemical design and discovery. We are working to further improve *ChemSpaX* along diverse research lines. Improved force-field methods can definitely help improve the accuracy of *ChemSpaX*. A data-informed approach to estimate the hRMSD based on the identity of the skeleton molecule and functional groups placed can improve the predictive capabilities for screening applications. Parallelization of the code for placing functional groups is work in progress. Additionally, we are working on implementing a more flexible approach where the user can choose the frequency of intermediate optimizations with a higher-level method based on a predicted hRMSD threshold, and also get recommendations about optimal strategies which balance speed and accuracy.

*ChemSpaX* is aimed at accelerating chemical space exploration and we hope that it will bolster and democratize the efforts of the catalysis and molecular modelling communities towards data-driven discovery. *ChemSpaX* is free, open-source and will soon be available with an introductory Google collaboratory notebook which can be immediately used by researchers.

## Data availability

The *ChemSpaX* workflow is publicly available on our Github organization page: EPiCs-group (https://github.com/EPiCs-group). In addition to this manuscript, ESI† and all used datasets can be found *via*: DOI: 10.4121/14766345.

• full_datasets.zip contains datasets per investigated complex in Excel workbook format.

• geometry_files.zip contains geometry files for all structures in MDL Molfiles or *XYZ* format.

• Coulomb_matrix_HL_gap_Mn_pincers.zip contains names, Coulomb matrix representations, and DFT computed HOMO–LUMO gaps of Mn–CNC and Mn–PCP complexes based on FF geometries, and DFT optimized geometries.

## Author contributions

The code for ChemSpaX was written by A. V. K. and V. S. Generation of functionalized structures, the compilation of datasets, and execution and analysis of DFT & xTB calculations was performed by A. V. K. under supervision of V. S. Machine learning models were developed by V. S. E. A. P. and V. S. conceived the project. E. A. P. played an advisory role and directed the project. All the authors discussed the results and wrote the manuscript.

## Conflicts of interest

There are no conflicts of interest to declare.

## Supplementary Material

DD-001-D1DD00017A-s001
