# Supplementary material for: ChemSpaX: exploration of chemical space by automated functionalization of molecular scaffold
Source: Digit Discov. 2022 Jan 6;1(1):8–25. doi: 10.1039/d1dd00017a (PMC8887922; doi:10.1039/d1dd00017a)
Supplement: DD-001-D1DD00017A-s001 [file DD-001-D1DD00017A-s001.pdf]

Electronic Supplementary Material (ESI) for Digital Discovery.
This journal is © The Royal Society of Chemistry 2022

SUPPORTING INFORMATION

ChemSpaX: Exploration of chemical space by automated functionalization of molecular scaffold

Adarsh V. Kalikadien, Evgeny A. Pidko* and Vivek Sinha*

Inorganic Systems Engineering Group, Department of Chemical Engineering, Delft University of Technology, Van der Maasweg 9, 2629 HZ Delft, The Netherlands

Corresponding authors: Vivek Sinha (V.Sinha@tudelft.nl) and Evgeny A. Pidko (E.A.Pidko@tudelft.nl)

Table of Contents

S1. Remarks 3
S2. Calculation of the centroid vector and the rotation matrix 3
S3. Observed issues with FF optimization 5
S4. Comparison of calculated energies of reaction using xTB or DFT for pincer complexes 6
RuPNP 6
Mn-pincers 6
S5. Analysis of $\Delta\Delta \mathrm{EFF(HX)}$ for functionalized Mn-pincers 7
S6. Distribution of Gibbs free energy for RuPNP 8
S7. Distribution of hRMSD for pincer complexes 9
RuPNP 9
Mn-pincers 10
S8. hRMSD for Mn-PNN complexes 11
S9. Comparison of DFT and xTB calculated HOMO-LUMO gap for Pincer complexes 11
RuPNP 11
Mn-pincers 12
S10. Error propagation of HOMO-LUMO gap for Co porphyrins 14
S11. HOMO-LUMO gap prediction via OLS for functionalized Co porphyrins 15
S12. XGBoost regressor code for predicting DFT computed HOMO-LUMO gap of Mn-pincer 15
S13. Bibliography 16

S1. Remarks

With regards to computational methods following nomenclature is adopted:

GFN2-xTB(GAS): optimization using Grimme's xTB (6.3.3) package.

GFN2-xTB(THF): optimization and hessian calculation in THF using the GBSA solvation model using Grimme's xTB (6.3.3) package.

BP86(GAS): DFT calculations were performed at various levels of theory. Geometry optimizations were performed in the gas phase using BP86 XC functional and def2-SVP basis set. Free energy corrections were obtained via hessian calculations within the harmonic approximation at the same level of theory. This method is denoted as BP86(GAS).

PBE1PBE(thf) (or PBE0(THF)) and BP86(THF): To include the solvent effects the electronic energies were further refined via single point energy calculations using SMD solvation method with THF as solvent. Two XC functionals namely BP86 and PBE1PBE were used with a triple zeta quality basis set (def2-TZVP). The free energy corrections obtained via hessian calculations at BP86(gas) level of theory were added to the electronic energies obtained during the singlepoint energy calculations. These methods are denoted as PBE0(THF) and BP86(THF).

We have calculated pearson correlation coefficient (R^2) related to a linear fitting ($\hat{y_i} = ax_i + b$) for all case where two methods/quantities (x_i, y_i) were compared in a scatter plot. We computed the root mean squared error (RMSE) related to the linear fit as :

$$RMSE = \sqrt{\frac{\sum_{i=1}^{N}(\hat{y_i} - y_i)^2}{N}}$$

The XYZ files of structures and datasets used for this publication are attached.

ChemSpaX is publicly available on our group's Github page (https://github.com/EPiCs-group/) together with a manual.

S2. Calculation of the centroid vector and the rotation matrix

The centroid vector starts at the central atom of the substituent group and points towards the centroid of the shape (triangle in the case of a tetrahedral substituent) formed by the atoms at the edges. In ChemSpaX an automated calculation of this centroid vector is done per substituent group. This can be visualized using a hypothetical tetrahedral substituent (Figure 1). The asymmetrical substituent is transformed into a hypothetical symmetrical tetrahedral substituent, where the centroid (yellow) is used to calculate the centroid vector (Figure 1, top). This centroid vector is then used to rotate and translate the whole substituent group.

The correct rotation matrix is determined by two unit vectors, 1) the bond that will be functionalized (a unit vector **a** between *bonded_atom* and *atom_to_be_functionalized*) and 2) the substituent's centroid vector (**b**). The mathematical details are described in a post on the Mathematics Stack Exchange [10]. A rotation matrix (**R**) rotates unit vector **a** onto unit vector **b**. Let **v** = **a** × **b**, $s = \|v\|$ (sine of angle) and **c** = **a**·**b** (cosine of angle) [11]. **R** is given by

$$\boldsymbol{R} = I + [v]_x + [v]_x^2 \frac{1-c}{s^2}, c \neq -1$$

$$\boldsymbol{R} = I, c = -1$$

Where $[\boldsymbol{v}]_x$ is the skew-symmatric cross-product matrix of **v**.

$$[\boldsymbol{v}]_x = \begin{pmatrix} \mathbf{0} & -\boldsymbol{v_3} & \boldsymbol{v_2} \\ \boldsymbol{v_3} & \mathbf{0} & -\boldsymbol{v_1} \\ -\boldsymbol{v_2} & \boldsymbol{v_1} & \mathbf{0} \end{pmatrix}$$

Using

$$\frac{1-c}{s^2} = \frac{1-c}{1-c^2} = \frac{1}{1+c}$$

Gives

$$\boldsymbol{R} = I + [\boldsymbol{v}]_x + [\boldsymbol{v}]_x^2 \frac{1}{1+c}, \qquad c \neq -1$$

The rotation matrix (**R**) is applied to the whole substituent group, followed by a translation of the whole group to the specified bonding distance from the skeleton. The complete workflow is summarized in the bottom part of figure 1.

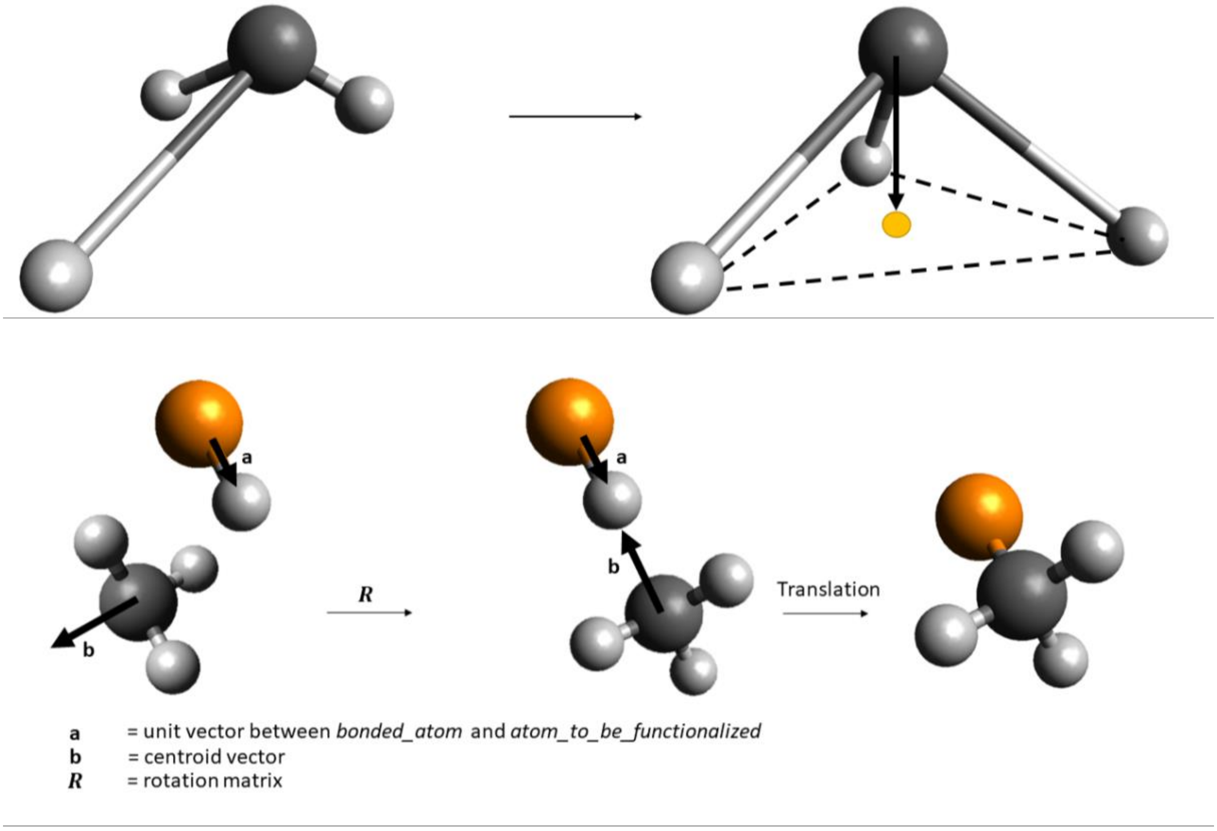

Figure 1. (top) Creation of a hypothetical symmetrical molecule and calculation of the centroid vector (bottom) Rotation and translation of a substituent group as implemented in ChemSpaX.

S3. Observed issues with FF optimization

When functionalizing a complex recursively, it was observed that using Openbabel's universal force field optimization (UFF) for geometry optimization would often misalign some of the hydrogen atoms. as illustrated in figure 2.

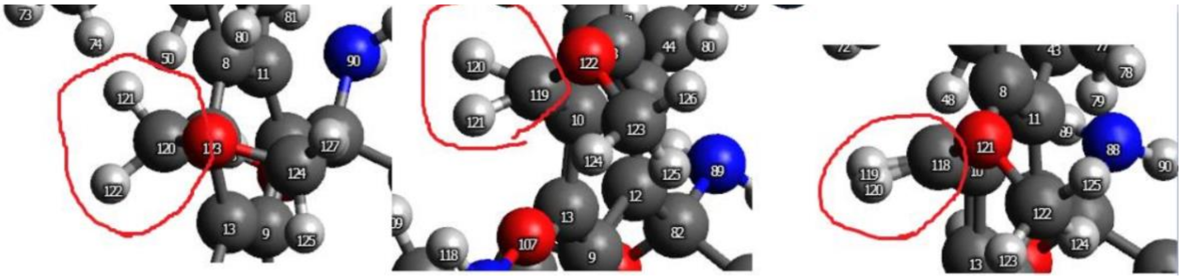

Figure 2. Misaligned hydrogen atoms during serial functionalization and optimization with UFF.

In the same scenario, but using Openbabel's GAFF only for geometry optimization, bonds between carbon and a halogen would have an incorrect angle. Which is shown in figure 3, with the incorrect angle on the right side and the correct angle on the left side of the figure.

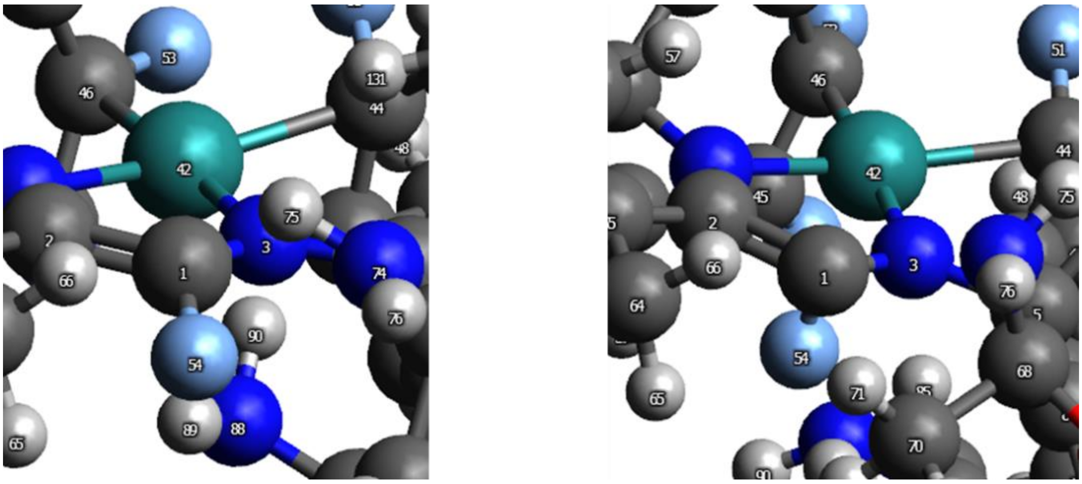

Figure 3. Incorrect angle (left) and correct angle of a CF bond when optimizing a TM complex with Openbabel's GAFF only.

Using a combination of GAFF and UFF for geometry optimization resulted in a highly increased probability of an error-free geometry.

S4. Comparison of calculated energies of reaction using xTB or DFT for pincer complexes

RuPNP

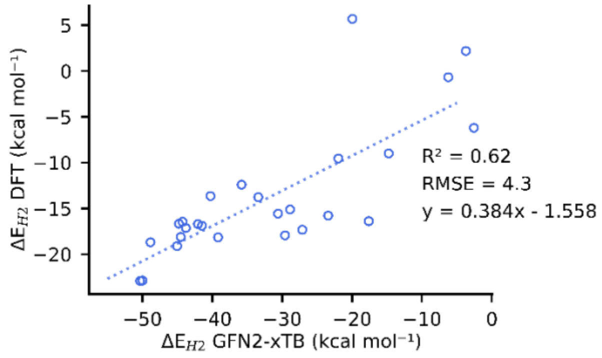

Figure 4. Comparison of ΔE calculated by BP86(GAS) vs GFN2-xTB(GAS).

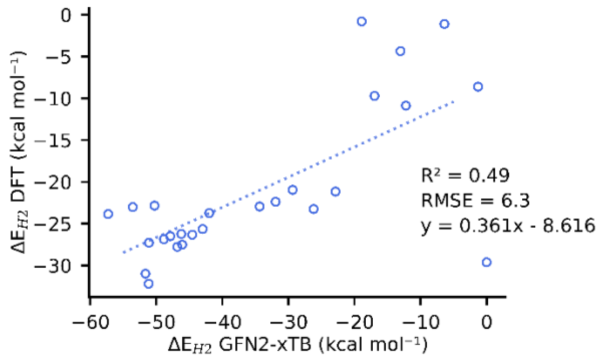

Figure 5. Comparison of ΔE calculated by PBE0(THF) vs GFN2-xTB(THF).

Mn-pincers

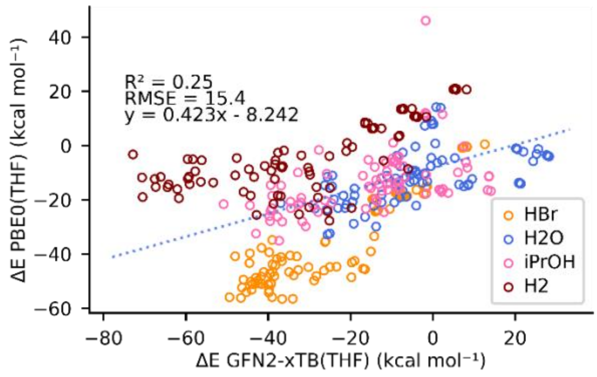

Figure 6. Comparison of ΔE calculated by PBE0(THF) vs GFN2-xTB(THF) for various adducts. The correlation is shown for the total x and y dataset.

Table 1. Individual Person's correlation coefficient (R^2) for the linear correlation between ΔE calculated by PBE0(THF) vs GFN2-xTB(THF) for various adducts.

**H-X**	**R^2**
HBr	0.83
H_2O	0.29
i-PrOH	0.27
H_2	0.53

S5. Analysis of $\Delta\Delta E_{FF}$(HX) for functionalized Mn-pincers

Ligand	Br	H	OH	i-PrO
PCP	17.10±4.76	10.80±7.25	20.96±9.36	25.60±23.45
PNN	36.37±16.83	28.05±18.42	63.28±36.79	56.06±32.05
CNC	8.94±8.08	12.16±9.64	8.31±10.97	16.92±22.83

Table 2. Mean and standard deviation ($\mu \pm \sigma 2$) for ΔΔEFF for various H-X and ligands in kcal mol−1 .

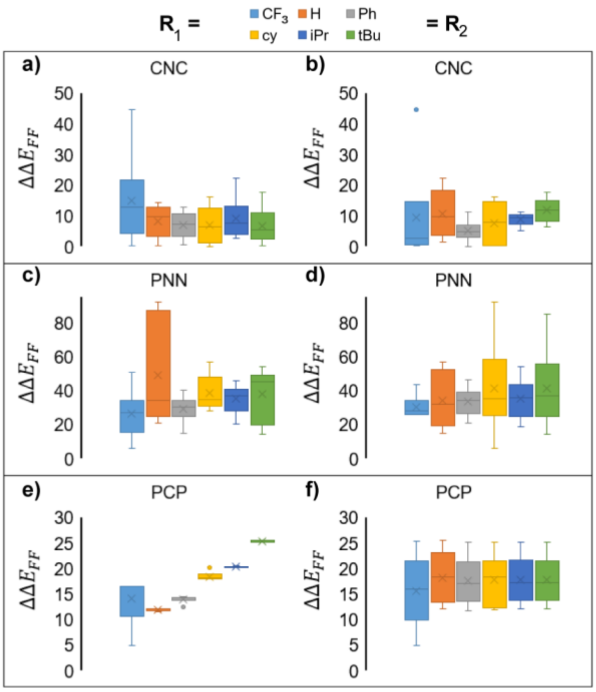

Figure 7. Box plots showing the distribution of $\Delta\Delta E_{FF}$ (kcal mol^{-1}) for reactive adsorption of H-X (X = Br) for (a,b) CNC (c,d) PNN and (d,e) PCP ligand based Mn complexes. For each ligand functionalization effect of the donor (R_1) and backbone (R_2) groups on the spread of $\Delta\Delta E_{FF}$ is visualized.

The distribution of $\Delta\Delta E_{FF}$ for the reactive adsorption of HBr for all the ligands and functionalization sites (R_1 and R_2) is shown in Figure 7. For the CNC ligand complexes variation of the functional group at the R_2 position with fixed R_1 (Figure 7a) led to similar $\mu(\Delta\Delta E_{FF}) < 10$ kcal mol^{-1} for all groups except for $R_1 = CF_3$. Since functionalization site R_2 is primarily a ligand backbone site, it seems that the effective relaxation of the ligand during DFT optimization is similar for all the CNC complexes. Variation of the functionalization group at R_1 results in a wider spread in the $\Delta\Delta E_{FF}$ values for CF_3, H and cy groups, while *i*-Pr substituents show most narrow distribution with $\mu(\Delta\Delta E_{FF}) = 9.01$ kcal mol^{-1}

. All PNN ligand complexes showed high and widespread μ($\Delta\Delta E_{FF}$) values. Most notable is R_1 = H which shows a rather large spread indicating large geometric relaxations when different ligands are introduced on the backbone sites. PCP ligand complexes are interesting for their almost linearly increasing μ($\Delta\Delta E_{FF}$) as the size of the functional group on the R_1 site is increased while the spread in the energies is minimal (Figure 7e). Furthermore, the variation at site R_2 shows an almost constant μ($\Delta\Delta E_{FF}$) with similar spread (Figure 7f). This observation indicates the source of spread is related to a larger geometric relaxation near the metal site during DFT based geometry optimization. Site R_2 being farther away from the metal center seems to have minimal and approximately constant impact on the geometries, and is rather well optimized by the force-field. We also compared the individual difference in energy between FF and DFT optimized geometries for M(X)-L(H) and M-L complexes. In general, we found that the energy of FF optimized M(Br)-L(H) complexes were closer to their DFT counterparts when compared to corresponding M-L complexes. This observation reveals that the M-L complexes have a larger contribution to the $\Delta\Delta E_{FF}$. This is consistent with the distribution of hRMSDs which are generally higher for M-L (pristine) complexes (see Figure 6 in the main text).

It must be noted here that the hRMSDs and $\Delta\Delta E_{FF}$ can be reduced by introducing intermediate optimization with a higher level of theory as discussed in the main text.

S6. Distribution of Gibbs free energy for RuPNP

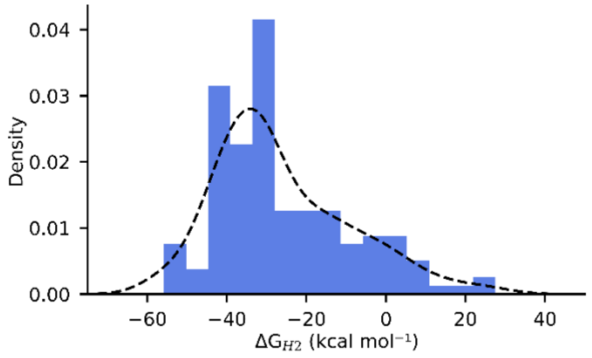

Figure 8. ΔG calculated by GFN2-xTB for all generated RuPNP geometries

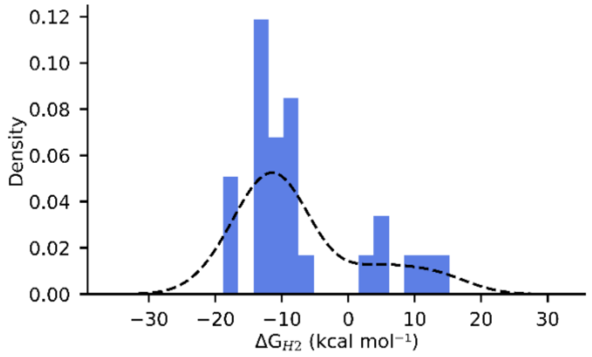

Figure 9. ΔG calculated using PBE0(THF) for 26 selected RuPNP geometries

S7. Distribution of hRMSD for pincer complexes

RuPNP

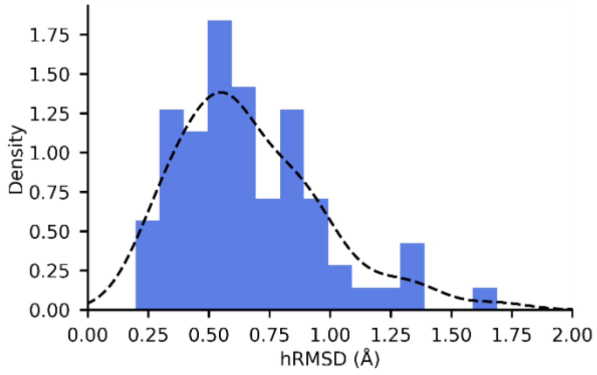

Figure 10. Distribution of hRMSD for ChemSpaX generated structures (newly placed substituents optimized with FF) compared against DFT (BP86) optimized structures.

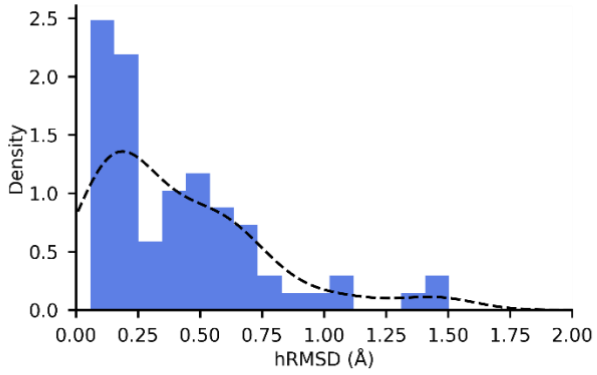

Figure 11. Distribution of hRMSD for GFN2-xTB optimized structures compared against DFT (BP86) optimized structures.

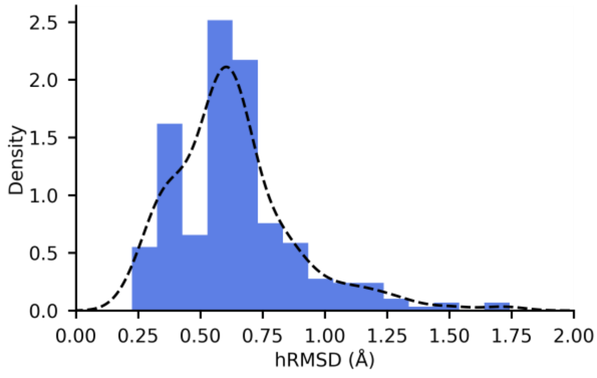

Figure 12. Distribution of hRMSD for ChemSpaX generated structures (newly placed substituents optimized with FF) compared against GFN2-xTB optimized structures.

Mn-pincers

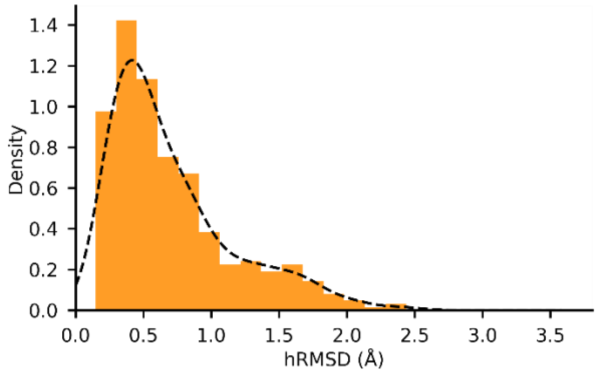

Figure 13. Distribution of hRMSD for ChemSpaX generated structures (newly placed substituents optimized with FF) compared against DFT (BP86(GAS)) optimized structures. Plotted for all datapoints.

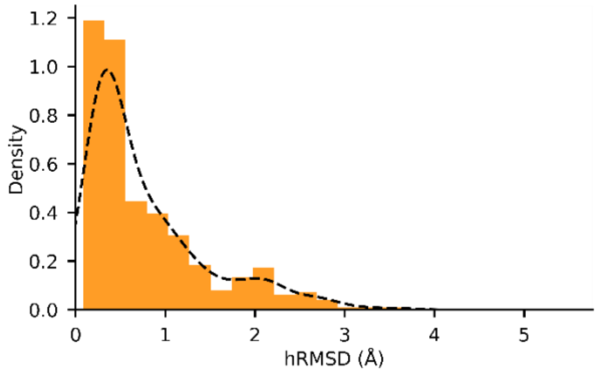

Figure 14. Distribution of hRMSD for GFN2-xTB (THF) optimized structures compared against DFT (BP86 (GAS)) optimized structures. Plotted for all datapoints.

S8. hRMSD for Mn-PNN complexes

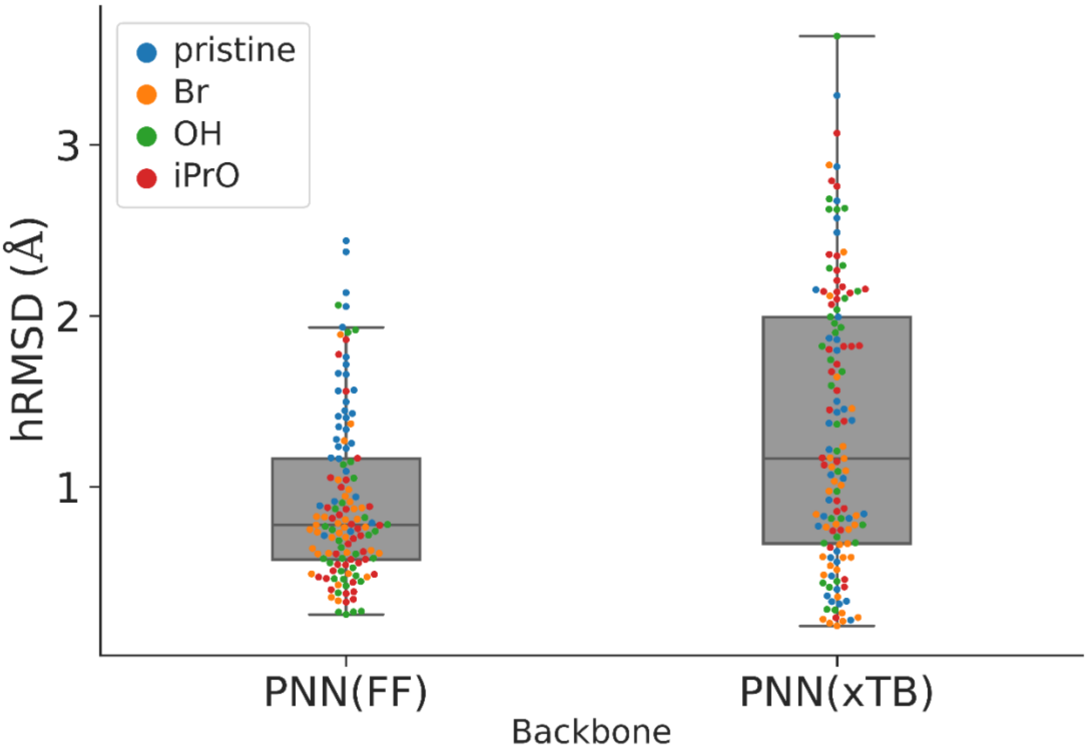

Figure 15. Distribution of hRMSD for Mn-PNN complexes computed against DFT based geometry optimization for (left) FF geometries and (right) xTB optimized geometries.

S9. Comparison of DFT and xTB calculated HOMO-LUMO gap for Pincer complexes

RuPNP

The HOMO-LUMO gap of the RuPNP pincers calculated for GFN2-xTB(THF) optimized geometries was compared to the HOMO-LUMO gap of BP86(THF) optimized structures. It was found that the HOMO-LUMO gap calculated by GFN2-xTB has a decent correlation with the DFT computed HOMO-LUMO. This result is shown in Figure where a R^2 of 0.74 and a RMSE of 0.4 eV was found. This indicates a reasonable accuracy of the GFN2-xTB calculated HOMO-LUMO gap, which can be useful in HTS applications for replacing resource-consuming DFT calculations.

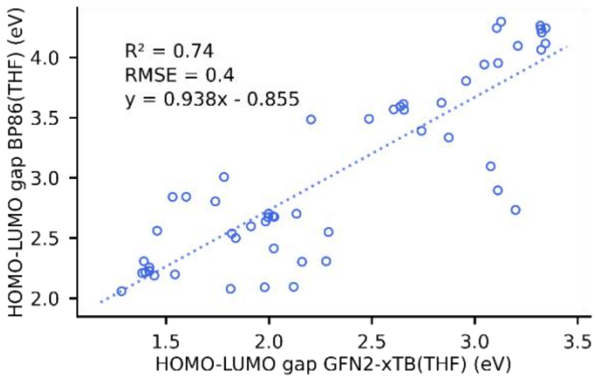

Figure 16. Comparison of BP86(THF) SP computed HOMO-LUMO gap on a DFT optimized geometry (y-axis) and a fully GFN2-xTB optimized geometry (x-axis).

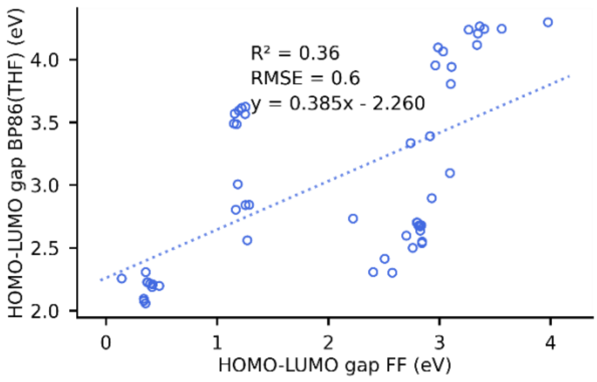

Figure 17. Comparison of BP86(THF) SP computed HOMO-LUMO gap on a FF optimized (x-axis) geometry, and a fully DFT optimized geometry (y-axis).

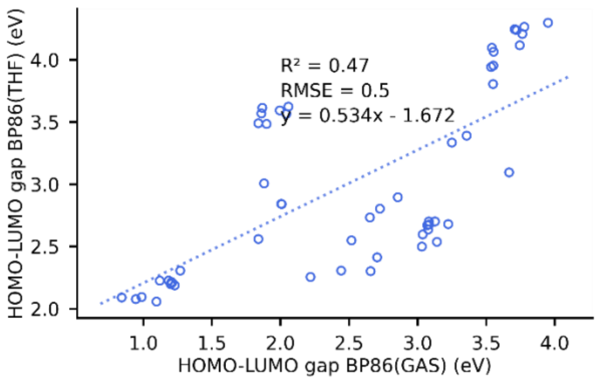

Figure 18. Comparison of HOMO-LUMO gap calculated using BP86(THF) and BP86(GAS), which shows the effect of solvation on the HOMO-LUMO gap.

Mn-pincers

For the Mn-pincers, the correlation between HOMO-LUMO gaps computed using GFN2-xTB(THF) and BP86(THF) was worse (R^2 = 0.3). The correlation for various analyzed adducts on the metal site and for the various backbones are shown below.

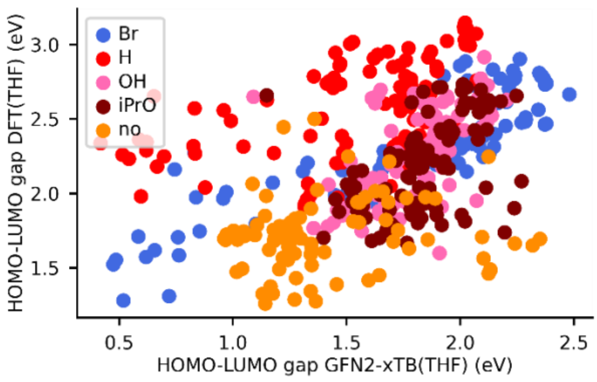

Figure 19. Comparison of HOMO-LUMO gap calculated by BP86(THF) against GFN2-xTB(THF) for various adducts on the metal site.

Table 3. Pearson's correlation coefficient and RMSE (calculated using the linear fit) of HOMO-LUMO gap by BP86(THF) against GFN2-xTB(THF) compared for various adducts on the metal site.

Adduct on metal site	R^2	RMSE (eV)
Br	0.74	0.18
H	0.26	0.28
OH	0.32	0.25
iPrO	0.23	0.27
no	0.008	0.23

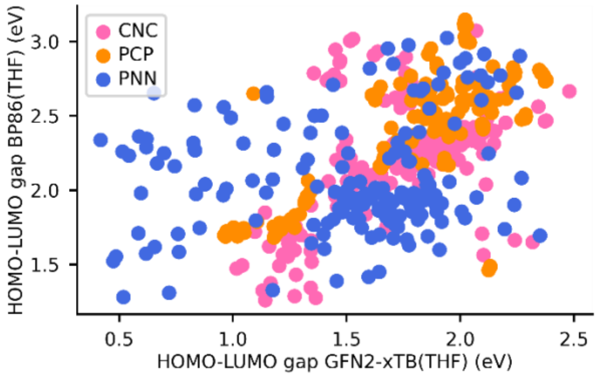

Figure 20. Pearson's correlation coefficient and RMSE (calculated using the linear fit) of HOMO-LUMO gap by BP86(THF) against GFN2-xTB(THF) *compared for various ligand backbones.*

Table 4. Pearson's correlation coefficient and RMSE of HOMO-LUMO gap comparison for various ligand backbones

Ligand backbone	R^2	RMSE (eV)
PCP	0.58	0.26
PNN	0.28	0.37
CNC	0.25	0.34

S10. Error propagation of HOMO-LUMO gap for Co porphyrins

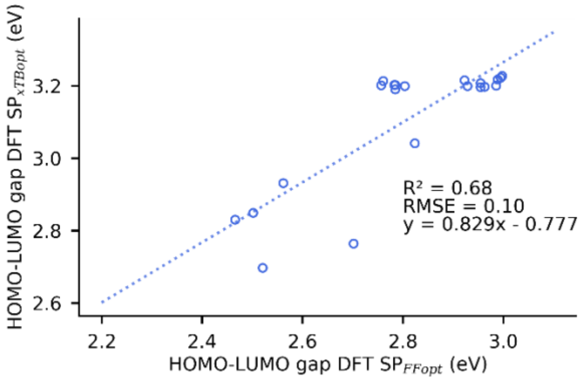

Figure 10. HOMO-LUMO gap computed using GFN2-xTB//PBE0(THF)-SP vs FF//PBE0(THF)-SP.

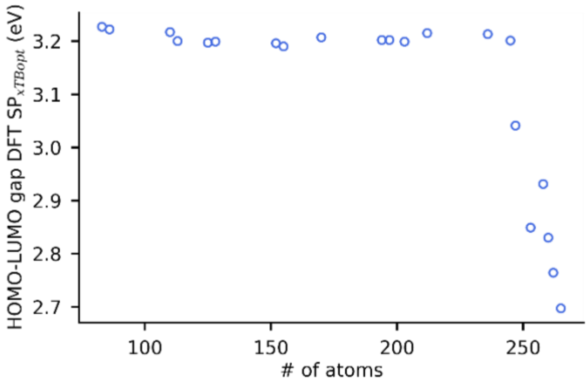

Figure 11. HOMO-LUMO gap computed using GFN2-xTB//PBE0(THF)-SP with increasing number of atoms from subsequent functionalizations.

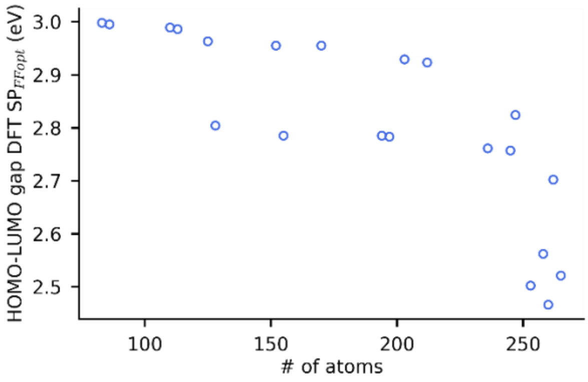

Figure 12. HOMO-LUMO gap computed using FF//PBE0(THF)-SP with increasing number of atoms from subsequent functionalizations.

S11. HOMO-LUMO gap prediction via OLS for functionalized Co porphyrins

The correlation between DFT and GFN2-xTB calculated HOMO-LUMO gaps for 280 selected structures was computed. Three features (1. number of atoms in the structure, 2. hRMSD (*ChemSpaX* generated FF versus GFN2-xTB structures) and 3. GFN2-xTB calculated HOMO-LUMO gap) were used to apply linear regression via OLS fitting and predict the DFT calculated HOMO-LUMO gap. These features were chosen to select relevant and easily computable features from xTB calculations for HTS applications. 75% of the dataset was applied to learn the DFT calculated HOMO-LUMO gap, 25% of the dataset was used for testing the model. It was observed when using the hRMSD as the only feature (training: R^2= 0.23, test: R^2 = 0.19) that this feature can be useful in more extensive machine learning methods. Its importance for electronic property prediction at DFT level of theory is currently not utilized by the simplicity of OLS.

Table 5. Weight per used feature in prediction of DFT calculated HOMO-LUMO gap using linear regression via OLS. Different combinations of features were used and a weight of 0 indicates that the feature was dropped. Pearson's correlation coefficient and RMSE for the training and test data are also shown.

Number of atoms	hRMSD	GFN2-xTB HOMO-LUMO gap	R^2 training	RMSE training	R^2 test	RMSE test
-0.15457	-0.0139	1.139595	0.81	0.09	0.71	0.12
-0.16277	0	1.140379	0.81	0.09	0.71	0.12
0	0	1.282884	0.77	0.1	0.71	0.12
0	-0.16434	1.188671	0.8	0.1	0.71	0.12
0	-0.46424	0	0.23	0.19	0.19	0.2

S12. XGBoost regressor code for predicting DFT computed HOMO-LUMO gap of Mn-pincer

```
import numpy as np

import pandas as pd

from sklearn.model_selection import train_test_split
```

```
from xgboost import XGBRegressor

# NOTE: Make sure that the outcome column is labeled 'target' in the data file
tpot_data = pd.read_csv('CM_HL_FF.csv', sep=',')
features = tpot_data.drop(['Name','HL'], axis=1)
training_features, testing_features, training_target, testing_target = \
            train_test_split(features, tpot_data['HL'], random_state=42)

# Average CV score on the training set was: -0.05452735277923859
exported_pipeline = XGBRegressor(alpha=1, learning_rate=0.1, max_depth=7, min_child_weight=15, n_estimators=100, n_jobs=1, objective="reg:squarederror", subsample=0.7500000000000001, tree_method="gpu_hist", verbosity=0)
# Fix random state in exported estimator
if hasattr(exported_pipeline, 'random_state'):
    setattr(exported_pipeline, 'random_state', 42)

exported_pipeline.fit(training_features, training_target)
results = np.array(exported_pipeline.predict(testing_features))
testing_target = np.array(testing_target)
train_results = np.array(exported_pipeline.predict(training_features))
training_target = np.array(training_target)
```

Complete dataset with coulomb matrix representations and DFT computed HOMO-LUMO gaps will be available via the 4TU database (https://doi.org/10.4121/14766345).

S13. Bibliography

[1] A. Kalikadien, "Automated data-driven exploration of chemical space for catalysts," 2021. [Online]. Available: http://resolver.tudelft.nl/uuid:cb84f7a8-8780-4813-85e9-f9a900f88270.

[2] J. van den Berg, "Calculate Rotation Matrix to align Vector A to Vector B in 3d? Mathematics Stack Exchange," 2016. [Online]. Available: https://math.stackexchange.com/q/476311.
